# Sutureless Scleral-Fixated Soleko Fil Carlevale Intraocular Lens and Associated Pars Plana Vitrectomy in Aphakia Management: A National Multicenter Audit

**DOI:** 10.3390/jcm14113963

**Published:** 2025-06-04

**Authors:** Lorena Ferrer-Alapont, Carolina Bernal-Morales, Manuel J. Navarro, Diego Ruiz-Casas, Claudia García-Arumí, Juan Manuel Cubero-Parra, Jose Vicente Dabad-Moreno, Daniel Velázquez-Villoria, Joaquín Marticorena, Julián Zarco-Bosquet, Félix Armada-Maresca, Cristina Irigoyen, Juan-Francisco Santamaría-Álvarez, Pablo Carnota-Méndez, Idaira Sánchez-Santos, Nuria Olivier-Pascual, Francisco Javier Ascaso, Javier Zarranz-Ventura

**Affiliations:** 1 Hospital Clínic of Barcelona, University of Barcelona, 08036 Barcelona, Spain; 2 August Pi i Sunyer Biomedical Research Institute (IDIBAPS), 08036 Barcelona, Spain; 3 Hospital Universitario Ramón y Cajal, 28034 Madrid, Spain; 4 Hospital Universitari Vall d’Hebron, 08035 Barcelona, Spain; 5 Hospital La Arruzafa, 14012 Córdoba, Spain; 6 Hospital Universitario La Paz, 28046 Madrid, Spain; 7 Clínica Villoria, 36001 Pontevedra, Spain; 8 Complejo Hospitalario Universitario de Santiago de Compostela, 15706 A Coruña, Spain; 9 Hospital de Sagunto, 46520 Valencia, Spain; 10 Hospital Universitario San Francisco de Asís, 28002 Madrid, Spain; 11 Hospital Universitario de Donostia, 20014 Donostia, Spain; 12 Hospital Universitario de Bellvitge, 08907 Barcelona, Spain; 13 Centro de Ojos de A Coruña, 15006 A Coruña, Spain; 14 Hospital de La Princesa, 28006 Madrid, Spain; 15 Complejo Hospitalario Universitario de Ferrol, 15405 A Coruña, Spain; 16 Hospital Clínico Universitario Lozano Blesa, 50009 Zaragoza, Spain

**Keywords:** aphakia, sutureless scleral-fixated IOL, posterior chamber IOL, trans-scleral, Carlevale, SSF Soleko Fil, pars plana vitrectomy

## Abstract

**Objective:** The aim of this study was to evaluate the clinical outcomes of sutureless scleral-fixated (SSF) Soleko Fil Carlevale intraocular lens (SC-IOL) implants associated with pars plana vitrectomy (PPV) in patients with aphakia secondary to complicated cataract surgery or IOL luxation nationwide. **Methods:** A multicenter, national, retrospective study of 268 eyes (268 patients) which underwent simultaneous PPV and SC-IOL implantation was conducted. Demographics; ocular data; pre-surgical, surgical and post-surgical details; and refractive results were collected. Intra- and postoperative complications and management details were described. Best-corrected visual acuity (BCVA), intraocular pressure (IOP) and central retinal thickness (CRT) were collected at 1 week and at 1, 3, 6 and 12 months post-surgery. Kaplan–Meier curves were constructed to assess the cumulative probability of postoperative BCVA, IOP levels, macular edema (ME) and corneal decompensation. **Results:** The cumulative probability of final VA ≤ 0.3 logMAR was 64.4% at 12 months follow-up. The probability of IOP > 21, ≥25 and ≥30 mmHg was 29.8%, 16.9% and 10.1%, respectively, and the cumulative probability of IOP-lowering treatment was 42.3% at 12 months. Glaucoma surgery was required in 3.7% of the eyes (10/268). The cumulative probability of postoperative ME development was 26.6% at 12 months, managed with topical treatment alone (73.5%) and intravitreal injections (26.5%). Corneal transplantation was required in 3.7% of the eyes (10/268). **Conclusions:** Sutureless scleral-fixated SC-IOL is an adequate therapeutic alternative in the management of aphakia with good visual results and an acceptable safety profile in routine clinical care. Longer-term studies are needed to evaluate its results and complications compared to other therapeutic alternatives.

## 1. Introduction

Cataract surgery is the most common surgical procedure worldwide due to demographic changes in the population and the increasing access to healthcare. The rise in cataract surgeries in recent years has consequently led to a greater number of patients with short- and long-term complications, including aphakia secondary to complicated cataract surgery and intraocular lens (IOL) luxation, respectively [1]. In scenarios of insufficient capsular support after cataract surgery or IOL dislocation, different surgical options for secondary IOL implantation arise, encompassing iris-claw IOLs, scleral-sutured IOLs and, more recently, sutureless scleral-fixated IOLs. Nevertheless, the management of aphakia in this context remains controversial, and no consensus exists about the ideal treatment technique in these cases, leading to decisions being made according to the surgeon’s personal preferences, the patient’s condition or IOL availability [1,2,3,4].

Sutureless scleral-fixated (SSF) lenses aim to eliminate complications related to iris fixation and those directly associated with suture ruptures in the mid- and long term [5]. Various techniques for SSF IOL fixation have been developed over the years, most of them using a three-piece IOL not specifically designed for intrascleral use, raising concerns about their long-term stability and centration [6,7,8].

The Soleko Fil Carlevale IOL (SC-IOL) is a recently introduced IOL for aphakia, designed specifically for SSF in the posterior chamber (PC). It is a foldable, single-piece IOL with flexible T-shaped harpoons that extend from the haptics to allow self-anchoring to the sclera without sutures. The advantages of this technique include a theoretically more anatomical and physiological IOL position in the PC, the absence of trauma to the iris, and its potential stability and centration [8,9,10,11].

In recent years, the use of SC-IOL has expanded in multiple countries, increasing real-world experience of their use. This expansion has led to multiple publications about the results and complications of SC-IOL, with most of these series being unicentric and having relatively short follow-ups [8,9,10,11,12,13,14,15,16,17,18,19,20,21,22,23,24,25,26]. For these reasons, studies with larger sample sizes and longer follow-up periods are still needed, particularly with regard to the analysis of associated short- and long-term complications. This multicenter audit was undertaken to evaluate the results and complications associated with SC-IOL in routine clinical care at a national level.

## 2. Materials and Methods

### 2.1. Study Design

This was a national, multicenter, observational retrospective study of consecutive case series involving SSF SC-IOL implantation with associated pars plana vitrectomy (PPV) following aphakia after complicated cataract surgery with no capsular support or IOL dislocation. A database was designed and an empty spreadsheet copy was distributed to the 14 participating centers. Data was collected locally at each center and returned in a pseudonymized manner using a consecutive coding method to be merged into a centralized national database, similarly to the previously completed study with another type of sutureless iris-fixated lenses (Artisan/Verisyse lenses) [27].

This study was approved by the institutional review board at the coordinator center of the study (CEIM, Hospital Clínic of Barcelona, study code HCB/2022/0527) and followed the tenets set forth in the Declaration of Helsinki.

### 2.2. Inclusion and Exclusion Criteria

Patients with aphakia secondary to IOL luxation or complicated cataract surgery with no capsular support who underwent SSF SC-IOL implantation and associated PPV were collected. Exclusion criteria included patients who underwent anterior vitrectomy instead of PPV and the implantation of other specific lenses for aphakia. In total, 290 eyes were selected, from which 11 eyes were excluded due to severe trauma with severe globe rupture and intraocular material loss (*n* = 4), IOL replacement due to Uveitis–Glaucoma–Hyphema Syndrome (*n* = 6) and chronic rhegmatogenous retinal detachment (*n* = 1). In bilateral cases (*n* = 11), only the first operated eye of each patient was included, and 11 eyes were excluded from the analysis. Finally, a total number of 268 eyes (268 patients) were included in the analysis.

### 2.3. Data Collection

Pre-surgical details included demographics, indication for surgery (complicated cataract surgery or IOL luxation), previous ocular history, best-corrected visual acuity (BCVA), ocular biometric data and refraction, slit lamp examination, intraocular pressure (IOP) and central retinal thickness (CRT) measured by optical coherence tomography (OCT). The surgical and intraoperative data collected included the PPV caliber setting (23G/25G/27G), scleral management (flaps/pockets), IOL power and intraoperative complications. Postoperative information included BCVA, slit lamp examination, IOP, CRT and treatment required at 1 week and at 1, 3, 6 and 12 months post-surgery. Postoperative complications and their management details were also collected. Macular edema (ME) was defined as CRT > 300 microns, and corneal decompensation was determined at the physician’s discretion, as endothelial cell counts were not conducted in daily routine examinations. No missing values were substituted in cases with incomplete data.

### 2.4. Surgical Technique

Standard complete PPV was completed in all cases (100%) using 23G and 25G systems, and SC-IOL implantation was performed locally at the physician’s discretion, with minor surgical variations, according to the individual criteria of each surgeon. These surgical variations included the use of scleral flaps, scleral pockets, anchoring via 25G sclerotomy at 2.0 or 2.5 mm from the limbus, and the use of a clear cornea or scleral tunnel incision, as well as different periocular anesthetic methods (sub-Tenon’s or peribulbar injections). In all cases, the T-haptic was secured using a 25G sclerotomy, and scleral flaps were closed with sutures, with scleral pockets sutured only in selected cases, according to the surgeon’s preference. No fibrin glue was used in any case. The implanted IOL was the PC SSF Soleko Fil Carlevale IOL (Soleko SPA, Pontecorvo, Italia) in all patients, with a biometric A-constant of 118.

### 2.5. Statistical Analysis

Statistical analysis was performed using SPSS 5.0 software (IBM SPSS Statistics v25.0; Armonk, NY, USA; IBM Corp.). For qualitative variable descriptive statistics, frequency and chi-square tests were used, considering 95% CIs (*p* = 0.05) for all proportions. The values of the continuous quantitative variables were expressed as means ± standard deviations (SDs) or medians and interquartile ranges accompanied by the statistical significance. Pre- and post-surgical changes were compared and analyzed to verify if there were statistically significant differences. Visual acuity measured in the Snellen notation was converted to Logarithm of the Minimum Angle of Resolution (LogMAR) equivalents for the purposes of statistical analysis. The cumulative probabilities of events (i.e., BCVA levels, IOP elevation, IOP-lowering treatment, ME development and resolution) occurring after IOL implantation were plotted as survival curves using the Kaplan–Meier (K-M) method and compared using the log-rank test. For all analyses, a *p*-value < 0.05 was considered statistically significant.

## 3. Results

A total of 268 eyes from 268 patients were included in the final study cohort. Surgeries were performed in 14 different hospitals across Spain (17 different surgeons). All the surgeries were performed by consultants, except for six surgeries (6/268, 2.2%) which were completed by fellows or senior trainees. A complete PPV was performed in all cases, using 23G in 35.1% of the cases and 25G in the other 64.9% of the eyes. Surgical variations between centers were minor, including the use of scleral flaps in 79.6% of the eyes while 20.4% used scleral pockets. Variations in flap sizes were also recorded, with 41.4% of surgeons performing 3 mm flaps, 15.2% 3.5 mm flaps, 42.4% 4 mm flaps and 1.0% 5 mm flaps. The main incision was performed in the clear cornea in 97.2% of the eyes. Table 1 shows the baseline characteristics, demographics and previous ocular conditions of the included eyes. The mean age was 70.9 ± 16.6 years, and 36.6% of the patients were female. At baseline, the mean preoperatory BCVA was 0.9 ± 0.6 logMAR, and the main indication for SC-IOL implantation was IOL luxation after previous uneventful cataract surgery (168 eyes, 62.7%) followed up by complicated cataract surgery (100 eyes, 37.3%).

### 3.1. Visual and Refractive Outcomes

**Figure 1:**
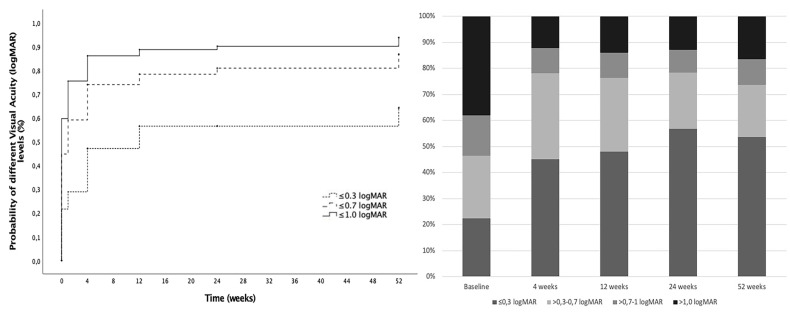
Visual acuity outcomes. (**Left**): Cumulative probability of achieving different visual acuity (VA) levels from baseline (dotted line: VA ≤ 0.3 LogMAR, dashed line: VA ≤ 0.7 LogMAR, solid line: VA ≤ 1.0 LogMAR). (**Right**): Distribution of eyes in each VA group at different timepoints during follow-up. (yellow: VA > 1.0 LogMAR, medium grey: VA > 0.7–1.0 LogMAR, orange: VA > 0.3–0.7, blue: VA ≤ 0.3 LogMAR).

From baseline to 52 weeks of follow-up, a significant improvement in mean BCVA was observed from 0.9 ± 0.6 logMAR (median: 0.8, IQR: 1.2) to 0.5 ± 0.5 (median: 0.3, IQR: 0.7) (*p* < 0.01) and remained significant at all timepoints. In the analysis by surgery indication subgroups, the mean BCVA at 52 weeks was 0.5 ± 0.5 logMAR (median: 0.2, IQR: 0.7) for the IOL luxation group and was 0.6 ± 0.5 (median: 0.4, IQR: 0.7) for the complicated cataract group, with no differences between groups (*p* = 0.47). Kaplan–Meier curves were generated to assess the cumulative probability of reaching various levels of BCVA (Figure 1, Table 2). At 12 months, the cumulative probability of BCVA ≤ 0.3 was 64.2%. The average spherical equivalent after surgery was 0.3 ± 1.3 diopters (D). In the group of eyes with both complete preoperative and postoperative refraction data (*n* = 115), the mean final astigmatism was −1.4 ± 1.2D. BCVA level (logMAR) evolution is presented in Table S1 (Supplementary Materials).

### 3.2. Intraocular Pressure Outcomes

**Figure 2:**
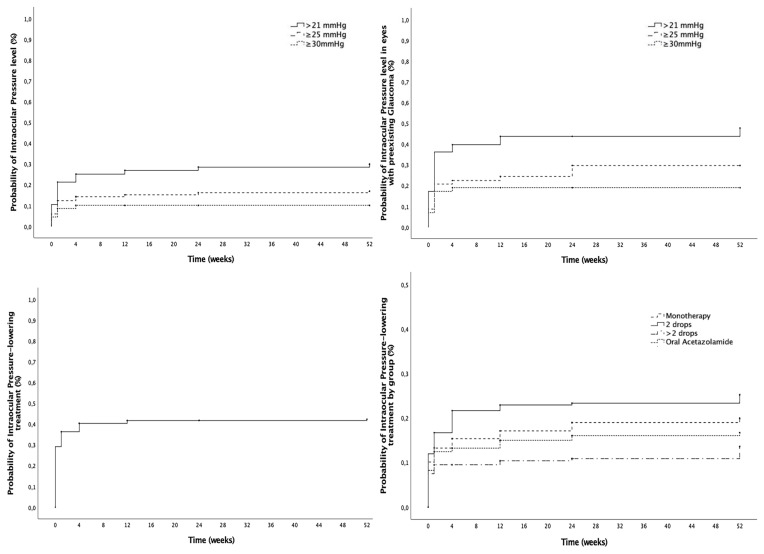
Intraocular pressure outcomes. (**Top-left**): Cumulative probability of different intraocular pressure (IOP) levels from baseline in the overall cohort (solid line: >21 mmHg, dashed line: ≥25 mmHg, dotted line: ≥30 mmHg). (**Top-right**): Cumulative probability of different intraocular pressure (IOP) levels from baseline in eyes with preexisting glaucoma (solid line: >21 mmHg, dashed line: ≥25 mmHg, dotted line: ≥30 mmHg). (**Bottom-left**): Cumulative probability of starting IOP-lowering treatment from baseline. (**Bottom-right**): Cumulative probability of starting IOP-lowering treatment from baseline by number of medications (solid line: monotherapy, dashed line: 2 different IOP-lowering drops, dotted line: more than 2 different drops, long-dashed line: oral acetazolamide).

The mean preoperative IOP was 17.3 ± 6.1 mmHg and lowered significantly after 12-month follow-up to 15.4 ± 3.9 mmHg (*p* = 0.02). At baseline, 13.7% (28/204) of the eyes already had an IOP > 21 mmHg prior to SC-IOL implantation. Figure 2 and Table 2 show the cumulative probability of reaching different IOP levels in the overall cohort and in eyes with preexisting glaucoma, as well as the probability and number of IOP-lowering medications, including topical drops and oral acetazolamide. At 12 months, the cumulative probability of IOP > 21 was 29.8%, and the cumulative probability of IOP-lowering treatment during follow-up at 52 weeks was 42.3%. Glaucoma surgery was undergone in 3.7% of the eyes studied (10/268). Among these, 90% (9/10) had preexisting glaucoma at baseline before the SC-IOL implantation. The IOP level (mmHg) evolution is presented in Table S2 (Supplementary Materials).

### 3.3. Macular Edema Development and Management

**Figure 3:**
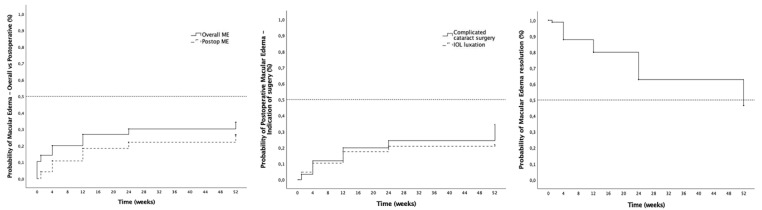
Macular edema (ME) and ME management outcomes. (**Left**): Cumulative probability of macular edema (ME) in the overall cohort (solid line) and postoperative ME (dashed line). (**Middle**): Cumulative probability of postoperative ME by indication for surgery groups after complicated cataract surgery (dashed line) and IOL luxation (solid line). (**Right**): Cumulative probability of ME resolution.

In eyes without preexistent ME, the cumulative probability of ME development at 12 months was 26.6%, with a higher cumulative probability of ME development in complicated cataract surgery eyes (34.4%) compared to the IOL luxation group (21.9%), although differences were not statistically significant (*p* = 0.14). At baseline, 10.4% (28/268) of the studied eyes already had ME. Including these eyes, the overall cumulative probability of ME was 34.3% at the 12-month follow up (Figure 3 and Table 2). Of the patients who developed ME during follow-up, 17.6% had diabetes. Of those, 53.8% had no signs of diabetic retinopathy, 15.4% had mild non-proliferative diabetic retinopathy (NPDR), 15.4% had moderate NPDR and 15.4% had treated proliferative diabetic retinopathy. With regard to the management of ME eyes (*n* = 83), topical treatment alone was administered in 73.5% (61/83) and intravitreal injections were performed in 26.5% (22/83) of the eyes during the 12 months follow-up, either with dexamethasone implants (13.3%, 11/83), anti-VEGF drugs (8.4%, 7/83) or both (4.8%, 4/83). With regard to safety, in four eyes treated with dexamethasone implants (4/11, 36.4%), implant migration to the anterior chamber was described and required surgical removal. The overall probability of ME resolution was 53.5% at 12 months (Figure 3), with a mean time to ME resolution of 15.8 ± 14.0 weeks after ME development. Lastly, the recurrence rate of ME after initial resolution was 20% at 12 months.

### 3.4. Corneal Complications

**Figure 4:**
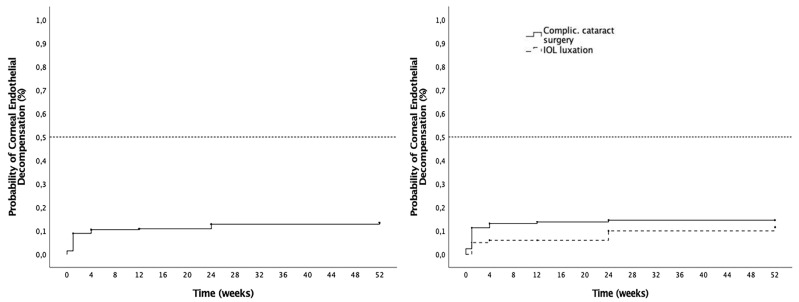
Corneal endothelial decompensation outcomes. (**Left**): Cumulative probability of corneal endothelial decompensation in the overall cohort. (**Right**): Cumulative probability of corneal endothelial decompensation in the overall cohort by indication for surgery groups, complicated cataract surgery (dashed line) and IOL luxation (solid line).

The cumulative probability of corneal endothelial decompensation was 13.4% at 12 months, and no significant differences (*p* = 0.28) were found between the complicated cataract surgery subgroup (11.6%) and the IOL luxation subgroup (14.6%) (Figure 4, Table 1). Corneal transplantation was required in 3.7% of the eyes (10/268), including Descemet Membrane Endothelial Keratoplasty (DMEK) in 40% (4/10) and Descemet Stripping Automated Endothelial Keratoplasty (DSAEK) in 60% (6/10). One DSAEK surgery was a re-DSAEK (first surgery before SSF SC-IOL implantation). Additionally, two patients required re-transplantation with Penetrating Keratoplasty (PK) after first DSAEK failure.

### 3.5. Other Complications

Other complications are shown in Table 3. The most common complication was haptic rupture or IOL disenclavation in eight cases (3.0%), followed by iris or ciliary body hemorrhage in five eyes (1.9%), vitreous hemorrhage in five cases (1.9%), retinal detachment in four eyes (1.5%) and localized peripheral choroidal hemorrhage in three eyes (1.1%). Among the overall cohort, two cases (0.7%) of reverse pupillary block were reported and resolved after completion of peripheral iridotomy (PI) with YAG laser treatment. Surgical PI was performed intraoperatively in 11 eyes (4.1%), among which no cases of pupillary block were described. No cases of endophthalmitis were reported. Secondary PPV was performed in nine cases (3.4%) due to retinal detachment (33.3%, 3/9), vitreous hemorrhage (22.2%, 2/9), epiretinal membrane (22.2%, 2/9) and broken haptics (2/9, 22.2%). IOL opacification, a long-term complication secondary to the hydrophilic nature of these IOLs, occurred in 0.7% of the eyes.

## 4. Discussion

This study provides real-world clinical outcomes and complication rates observed with SC-IOL implantation and associated PPV in a large multicenter series of aphakic eyes secondary to complicated cataract surgery or IOL luxation without capsular support.

Our national multicenter audit results show a significant improvement in BCVA from baseline, with 64.2% of the study eyes reaching a cumulative probability of BCVA ≤ 0.3 logMAR at 12 months. The mean final BCVA at 6-month and 12-month follow-up was 0.5 ± 0.5 logMAR for both timepoints, in line with previous reports from smaller series with shorter follow-ups [7,11,12]. Georgalas et al. reported better postoperative visual outcomes in a cohort where most of the eyes had previous IOL dislocation, suggesting that SC-IOL indication (IOL dislocation vs. complicated cataract surgery) might influence visual results [8]. Nevertheless, no significant differences in BCVA outcomes were found in our series when comparing IOL luxation and complicated cataract groups. It should be noted that the biometric calculations in both scenarios are particularly challenging with the SC-IOL and may have an impact on the final visual outcomes, in addition to other IOL-specific related factors such as IOL tilt. Recent comparative studies of SC-IOL and other aphakia techniques, such as iris-claw IOL, showed similar visual acuity outcomes, although SC-IOL was reported to have better refractive results [28,29].

Mean IOP change during follow-up was also assessed in our study. Interestingly, postoperative IOP significantly improved at 12 months after surgery compared to baseline. These results are consistent with what has been previously reported in other studies, where IOP did not significantly change before or after surgery or where there was even some degree of postoperative hypotony [8,12,18,20]. At 12 months, the cumulative probability of IOP > 21 was 29.8% and was slightly higher in those patients with pre-existing glaucoma. Likewise, 90% (9/10) of the studied eyes that required glaucoma surgery had a previous diagnosis of this condition. These two findings suggest that special care should be dedicated to glaucomatous eyes in order to minimize potential IOP spikes in the perioperative period secondary to the SSF SC-IOL implantation itself and to the potential impact of performing a complete PPV. In most cases, ocular hypertension could be managed with IOP-lowering drops without further significance, and the cumulative probability of using IOP-lowering treatment during follow-up was 42.3%. Peripheral iridotomy was performed during surgery in 11 eyes (4.1%), and 2 eyes with no previous PI developed a reverse pupillary block after surgery, a previously described complication of the SSF SC-IOL [30], which resolved after performing the PI. Due to the relatively low percentage of eyes that presented this complication, we do believe that performing an intraoperative PI during SC-IOL implantation should be considered only on a case-by-case basis to prevent postoperative reverse pupillary block. Moreover, the development of ciliary body complications and postoperative hypotony are underreported topics that require further research.

The macular edema rates reported in our study were higher than those previously described in the literature. We found an overall cumulative probability of ME development of 34.3% at 12 months, with 10.4% of eyes presenting ME at baseline, before the SSL SC-IOL implantation. Excluding patients with baseline ME, the cumulative probability of postoperative ME development was 26.6%, still higher than in other cohorts. These results differ from the highest rates of ME reported in the literature, which did not exceed 15%, such as the series published by Van Severen et al. with an ME rate of 14.9% [26], Vaiano et al. with a rate of 7.4% [12] and Rossi et al. with a rate of 5.1% [11]. These differences could be explained in several ways. On the one hand, some of these studies presented shorter follow-up times, which may have led to underestimation of late postoperative ME [18,26]. Furthermore, these cohorts were significantly smaller compared to the one in this study, such that the populations may have been insufficiently representative, resulting in a potential risk of publication bias. Another potential factor could be pseudoexfoliation, which was present in 27.6% of our cases and has shown controversial associations with ME, with some reports suggesting a higher risk [31] not confirmed by other authors [32]. Diabetes could have also been a confounding factor in the development of ME, as 17.6% of the patients who developed ME had this disease. And finally, there was a potential influence of surgery indication, with IOL luxation cases rather than complicated cataract surgery being predominantly included in the published data. Although we did not find differences between subgroups, previous reports suggest that IOL luxation may present lower ME development rates compared to complicated cataract surgery, which could have contributed to the findings observed [8,18]. While the multicenter nature of our study introduced a potential risk of variability in the results, this added to the external validity of the findings reported, as they reflect daily clinical practice at a national level. In our series, ME was managed successfully in many cases, and the cumulative probability of ME resolution was 53.5%, with 8.2% of eyes requiring intravitreal injections with dexamethasone implants or anti-VEGF. With regards to dexamethasone implants, 36.4% of the eyes had anterior chamber migration of the implant which required a new surgery for its removal, a consideration that should be taken into account in the ME treatment algorithm in these cases. Importantly, it should be noted that 20% ME recurrence was observed in eyes with previous ME resolution during the 12-month follow-up, hence the importance of longer follow-up times.

Postoperative corneal complications were also analyzed in our cohort. The cumulative probability of corneal endothelial decompensation was 13.4% at 12 months, without significant differences between SC-IOL surgery indication groups. This figure is consistent with other series, such as the study by Van Severen et al., which reported a corneal edema rate of 13.9% 1 month after surgery [26]. Transient corneal edema was also described in eyes with anterior chamber migration of a dexamethasone implant, which completely resolved after its surgical removal. Furthermore, SC-IOL may also be a therapeutic alternative in those patients with corneal decompensation who require corneal transplantation, since some series of combined surgeries have been published with acceptable results [33,34,35].

Among other complications, SC-IOL haptic rupture during surgical manipulation of the “T” haptics and/or intraoperative dislocation was the most common (3.0%), followed by iris or ciliary body hemorrhage and vitreous hemorrhage (both 1.9%). These figures are lower than those of previous reports, such as Rouhette et al.’s, which described damaged IOL (optic of haptics) in 12.5%, or [18] Vaiano et al.’s, which reported it in 11% [12] of cases, and similar to those of others, such as Van Severen et al.’s, which reported haptic ruptures in 2% of their series [26]. Finally, Georgalas et al. reported a rate of 5.3% of intraoperative IOL subluxation that was immediately resolved [8]. These intraoperative complications were probably related to the technical skills, intraoperative manipulation of the lens and the learning curve that SC-IOL implantation involves. Importantly, no cases of endophthalmitis were described in our series.

Our study has the limitations of being a retrospective study, where some data could have been missing, as it was not registered in medical records as part of the routine clinical care and is therefore not retrievable, for example, endothelial cell counts or specific techniques for IOL handling prior to IOL anchoring (i.e., in anterior chambers or vitreous cavities). Nonetheless, this series provides further knowledge about SC-IOL in a large group of patients with greater follow-up than previous studies. Moreover, the multicenter design of the study offers a wider picture of SC-IOL implantation in a single country, showing the variability between surgeons and centers and, consequently, providing more representative results of the current clinical practice in routine clinical care.

The results described in this study support the use of SC-IOL as a good therapeutic alternative for the management of aphakia, with adequate results in terms of visual acuity gains, though there were complications which have to be considered both at the time of surgery indication and during the postoperative follow-up. The most frequent intraoperative complications were related to the surgical technique and the IOL material, and postoperative complications included macular edema and IOP problems, mainly in patients with preexisting glaucoma and corneal decompensation, which have to be routinely evaluated as they could manifest months after surgery, highlighting the need for longer-follow-up series. The well-known benefits of SC-IOL include its sutureless nature, its great stability and long-term self-centration, which need to be evaluated jointly with the safety profile described above in individual case-by-case discussions with patients to inform treatment decisions. Further studies are required to analyze the long-term performance of this relatively novel IOL in comparison with other aphakia techniques or IOLs in larger cohorts in the near future.

## Figures and Tables

**Table 1:** Baseline characteristics of study eyes.

	Total	IOL Luxation	Complicated Cataract Surgery	*p*-Value
N (%)	268	62.7% (168/268)	37.3% (100/268)	-
Gender				
Female (%)	36.6 (98/268)	35.7 (60/168)	38.0 (38/100)	0.77
Laterality				
Right eye (%)	53.4 (143/268)	54.2 (91/168)	52.0 (52/100)	0.66
Age				
Mean ± SD	70.9 ± 16.6	72.5 ± 13.5	68.1 ± 20.5	0.06
Median (IQR)	75.0; 18.8	75.0; 15.5	75.0; 25.5	
Preop VA (logMAR)		0		
Mean ± SD	0.9 ± 0.6	0.9 ± 0.6	1.0 ± 0.6	0.02
Median (IQR)	0.8; 1.2	0.7; 1.3	1.0; 1.2	
IOP (mmHg)				
Mean ± SD	17.3 ± 6.1	17.3 ± 5.1	17.2 ± 7.6	0.86
Median (IQR)	16.0; 6.0	16.6; 6.0	15.0; 4.5	
Axial length (mm)				
Mean ± SD	24.5 ± 2.6	25.1 ± 3.0	23.6 ± 1.5	<0.01
Median (IQR)	23.7; 1.7	23.9; 2.3	23.5; 1.4	
Anterior chamber depth (mm)				<0.01
Mean ± SD	3.9 ± 0.9	4.4 ± 0.8	3.3 ± 0.7	
Median (IQR)	3.9; 1.4	4.3; 1.0	3.1; 1.1	
Preop macular edema	10.4 (28/268)	11.3 (19/168)	9.0 (9/100)	0.68
Pseudoexfoliation	27.6 (74/268)	30.4 (51/168)	23.0 (23/100)	0.12
Glaucoma	21.6 (58/268)	23.8 (10/168)	18.0 (18/100)	0.25
Diabetes	12.7 (34/268)	11.9 (20/168)	14.0 (14/100)	0.64
Diabetic retinopathy	6.0 (16/268)	7.1 (12/168)	4.0 (4/100)	0.17
High myopia	17.2 (46/268)	22.0 (37/168)	9.0 (9/100)	<0.01
Uveitis	6.0 (16/268)	6.0 (10/168)	6.0 (6/100)	0.99
Traumatism	14.2 (38/268)	12.5 (21/168)	17.0 (17/100)	0.32

**Table 2:** Cumulative probabilities of different events during follow-up.

	Baseline	1 Month	3 Months	6 Months	12 Months
Data entries at individual timepoints	*n* = 268	*n* = 206	*n* = 177	*n* = 139	*n* = 121
Visual acuity levels (logMAR)					
≤0.3 (%)	21.6	47.1	56.5	56.5	64.2
≤0.7 (%)	44.8	73.9	78.3	80.9	86.7
≤1 (%)	59.7	86.1	88.7	90.1	93.7
	*n* = 268	*n* = 206	*n* = 177	*n* = 139	*n* = 121
Cumulative probability of IOP levels (mmHg)					
>21 (%)	10.4	25.1	26.9	28.5	29.8
≥25 (%)	6.0	14.2	15.1	16.2	16.9
≥30 (%)	4.5	10.1	10.1	10.1	10.1
	*n* = 204	*n* = 202	*n* = 165	*n* = 123	*n* = 100
Cumulative probability of IOP-lowering drops (%)	21.0	40.3	41.6	42.3	42.3
Monotherapy (%)	10.1	15.3	17.1	18.9	19.9
2 drops (%)	11.9	21.6	22.9	23.3	25.2
>2 drops (%)	7.5	9.4	10.3	10.8	13.5
Oral acetazolamide (%)	8.2	13.2	14.9	16.0	16.7
	*n* = 212	*n* = 188	*n* = 165	*n* = 127	*n* = 97
Cumulative probability of macular edema development (%)	10.4	20.1	26.9	30.2	34.3
Cumulative probability of postoperative macular edema development (%)	-	10.8	18.3	22.1	26.6
Cumulative probability of macular edema resolution (%)	-	12.2	20.0	37.2	53.5
	*n* = 268	*n* = 155	*n* = 135	*n* = 113	*n* = 98
Cumulative probability of endothelial decompensation (%)	-	10.5	10.9	12.8	13.4
		*n* = 182	*n* = 156	*n* = 121	*n* = 94

**Table 3:** Other complications related to Carlevale implantation.

	Total
“T” Haptic rupture/IOL disenclavation	8 (3.0%)
Iris/ciliary body hemorrhage	5 (1.9%)
Vitreous hemorrhage	5 (1.9%)
Retinal detachment	4 (1.5%)
Choroidal hemorrhage	3 (1.1%)
IOL rotation/IOL upside down	3 (1.1%)
Hypotony	2 (0.7%)
Retinal tear	2 (0.7%)
Reverse pupillary block	2 (0.7%)
IOL opacification	2 (0.7%)
Haptic extrusion	2 (0.7%)
Sclerotomy leak	1 (0.4%)
Endophthalmitis	0 (0.0%)

## Data Availability

Data will be made available upon request from the corresponding author. The data are not publicly available due to privacy.

## Supplementary Materials

The following supporting information can be downloaded at https://www.mdpi.com/article/10.3390/jcm14113963/s1, Table S1: BCVA levels (logMar) evolution; Table S2: IOP levels (mmHg) evolution.

## Abbreviations

The following abbreviations are used in this manuscript:

SSF	Sutureless scleral-fixated
SC-IOL	Soleko Fil Carlevale intraocular lens
PPV	Pars plana vitrectomy
BCVA	Best-corrected visual acuity
IOP	Intraocular pressure
ME	Macular edema

## Appendix A

*Sutureless Scleral-Fixated Soleko Fil Carlevale IOL Spanish study group*
*(centers and principal investigators):*
**Hospital Clínic of Barcelona, University of Barcelona, Barcelona, Spain**
Javier Zarranz-Ventura, Lorena Ferrer-Alapont, Carolina Bernal-Morales, Manuel J. Navarro
**Hospital Ramón y Cajal, Madrid, Spain**
Diego Ruiz-Casas, Pablo de Arriba, Carolina Arruabarrena
**Hospital Universitari Vall d’Hebron, Barcelona, Spain**
Claudia García-Arumí, Olaia Subirá, Laura Sanchez-Vela, Jose García-Arumí
**Hospital La Arruzafa, Córdoba, Spain**
Juan Manuel Cubero-Parra, Consuelo Spínola, Mercedes Giménez de Azcárate, Juan Manuel Laborda
**Hospital Universitario La Paz, Madrid, Spain**
Jose Vicente Dabad-Moreno, Mónica Asencio-Duran, Miguel Del Piñal Alvarez de Buergo, Adriana de la Hoz Polo, María Capote Diaz, Almudena del Hierro Zarzuelo, Lucas San Juan Riera, Javier Coca Robinot, Ana Boto de los Bueis, Oriana D’Anna Mardero, Mireia Ariadna Roca Cabau, Maria del Pino Cidad, Felix Armadá-Maresca
**Clínica Villoria, Pontevedra, Spain**
Daniel Velázquez-Villoria, Alvaro Velazquez-Villoria, Rolando Herrera-Vasquez, Alejandra María Parra-Morales
**Complejo Hospitalario Universitario de Santiago de Compostela, A Coruña, Spain**
Joaquin Marticorena-Salinero, Maria Jose Blanco Teijeiro, Purificación Mera Yáñez
**Hospital de Sagunto, Valencia, Spain**
Julian Zarco-Bosquet, Hector Mascaros Mena, Raquel Burggraaf Sanchez de las Matas
**Hospital Universitario San Francisco de Asis, Madrid, Spain**
Felix Armadá-Maresca, Ana Boto de los Bueis
**Hospital Universitario de Donostia, Donostia, Spain**
Cristina Irigoyen-Laborra, Jorge Sánchez-Molina
**Hospital Universitario de Bellvitge, Barcelona, Spain**
Juan Francisco Santamaría-Alvarez, Lluis Arias-Barquet, Josep María Caminal, Estefanía Cobos, Pere García-Bru, Rahul Morwani, Daniel Lorenzo-Parra
**Centro de Ojos de A Coruña, A Coruña, Spain**
Pablo Carnota-Méndez, Carlos Méndez Vázquez, Carlos Ignacio Torres Borrego
**Hospital de La Princesa, Madrid, Spain**
Idaira Sanchez-Santos, Muxima Acebes García
**Complejo Hospitalario Universitario de Ferrol, A Coruña, Spain**
Nuria Olivier-Pascual, Rosa Arroyo Castillo
**Hospital Clínico Universitario Lozano Blesa, Zaragoza, Spain**
Francisco Javier Ascaso, Olivia Esteban Floria
