# Supplementary material for: Sutureless Scleral-Fixated Soleko Fil Carlevale Intraocular Lens and Associated Pars Plana Vitrectomy in Aphakia Management: A National Multicenter Audit
Source: J Clin Med. 2025 Jun 4;14(11):3963. doi: 10.3390/jcm14113963 (PMC12155773; doi:10.3390/jcm14113963)
Supplement: Supplementary file 1 [file jcm-14-03963-s001.zip › jcm-3648113-supplementary.pdf]

**SUPPLEMENTAL MATERIAL**

**Table S1.** BCVA levels (logMar) evolution

| | Baseline | 1 Month | 3 Months | 6 months | 12 months |
|---|---|---|---|---|---|
| Total (mean ± SD) | 0.9 ± 0.6 | 0.5 ± 0.5 | 0.5 ± 0.5 | 0.5 ± 0.5 | 0.5 ± 0.5 |
| (median; IQR) | 0.8; 1.2 | 0.4; 0.5 | 0.4; 0.5 | 0.3; 0.6 | 0.3; 0.7 |
| | n=268 | n=206 | n=177 | n=139 | n=121 |
| IOL-luxation group | 0.9± 0.6 | 0.5 ± 0.5 | 0.5 ± 0.5 | 0.5 ± 0.5 | 0.5 ± 0.5 |
| | 0.7; 1.3 | 0.4; 0.5 | 0.4;0.6 | 0.3; 0.6 | 0.2; 0.7 |
| | n=168 | n=132 | n=112 | n=87 | n=68 |
| Complicated cataract surgery group | 1.0 ± 0.6 | 0.5 ± 0.4 | 0.5 ± 0.4 | 0.5 ± 0.5 | 0.6 ± 0.5 |
| | 1.0; 1.2 | 0.5; 0.5 | 0.4; 0.5 | 0.3; 0.5 | 0.4; 0.7 |
| | n=100 | n=74 | n=65 | n=52 | n=53 |

**Table S2.** IOP levels (mmHg) evolution

| | Baseline | 1 Month | 3 Months | 6 months | 12 months |
|---|---|---|---|---|---|
| Total (mean ± SD) | 17.3 ± 6.1 | 16.3 ± 5.6 | 15.6 ± 5.0 | 15.7 ± 5.3 | 15.4 ± 3.9 |
| (median; IQR) | 16.0; 6.0 | 16.0; 5.0 | 15.0; 5.0 | 15.0; 4.0 | 16.0; 6.0 |
| | n=204 | n=202 | n=165 | n=123 | n=100 |
| IOL-luxation group | 17.3 ± 5.1 | 16.7 ± 5.8 | 16.0 ± 5.8 | 16.3 ± 6.2 | 15.01 ± 4.1 |
| | 16.0; 6.0 | 16.0; 4 | 15.0; 5.0 | 15.5; 4.0 | 14; 5.8 |
| | n=129 | n=128 | n=108 | n=78 | n=58 |
| Complicated cataract surgery group | 17.2 ± 7.6 | 15.6 ± 5.1 | 14.9 ± 3.1 | 14.6 ± 3.0 | 15.7 ± 3.6 |
| | 15.0; 4.5 | 15.0; 5.8 | 14.0; 6.0 | 14.0; 5.0 | 16.0; 3.8 |
| | n=75 | n=74 | n=57 | n=45 | n=42 |
